# FGF21 Mediates Mesenchymal Stem Cell Senescence via Regulation of Mitochondrial Dynamics

**DOI:** 10.1155/2019/4915149

**Published:** 2019-04-17

**Authors:** Xin Li, Yimei Hong, Haiwei He, Guojun Jiang, Wei You, Xiaoting Liang, Qingling Fu, Shuo Han, Qizhou Lian, Yuelin Zhang

**Affiliations:** ^1^Department of Emergency Medicine, Department of Emergency and Critical Care Medicine, Guangdong Provincial People's Hospital, Guangdong Academy of Medical Sciences, Guangzhou, Guangdong 510080, China; ^2^School of Medicine, South China University of Technology, Guangzhou, Guangdong 510515, China; ^3^Faculty of Pharmacy, Bengbu Medical College, Bengbu, Anhui 233000, China; ^4^Clinical Translational Medical Research Center, Shanghai East Hospital, Tongji University School of Medicine, Shanghai 200120, China; ^5^Otorhinolaryngology Hospital, The First Affiliated Hospital, Sun Yat-sen University, Guangzhou, Guangdong 510080, China; ^6^Department of Medicine, Li Ka Shing Faculty of Medicine, The University of Hong Kong, Hong Kong

## Abstract

Mesenchymal stem cell- (MSC-) based therapy is a novel strategy in regenerative medicine. The functional and regenerative capacities of MSCs decline with senescence. Nonetheless, the potential mechanisms that underlie their senescence are not fully understood. This study was aimed at exploring the potential mechanisms of fibroblast growth factor 21 (FGF21) in the regulation of MSC senescence. The senescence of MSCs was determined by senescence-associated *β*-galactosidase (SA-*β*-gal) staining. The morphology and the level of mitochondrial reactive oxygen species (ROS) of MSCs were assessed by MitoTracker and Mito-Sox staining, respectively. The expression of FGF21 and mitochondrial dynamics-related proteins was detected by Western blotting. As MSCs were expanded *in vitro*, the expression of FGF21 decreased. Depletion of FGF21 enhanced production of mitochondrial reactive oxidative species (ROS) and increased the senescence of early-passage MSCs whereas inhibition of ROS abolished these effects. The senescent MSCs exhibited increased mitochondrial fusion and decreased mitochondrial fission. Treatment of early-passage MSCs with FGF21 siRNA enhanced mitochondrial fusion and reduced mitochondrial fission. Moreover, treatment of mitofusin2- (Mfn2-) siRNA inhibited depletion of FGF21-induced MSC senescence. Furthermore, we demonstrated that depletion of FGF21-induced mitochondrial fusion was regulated by the AMPK signaling pathway. Treatment with an AMPK activator, AICAR, abrogated the depletion of FGF21-induced senescence of MSCs by inhibiting mitochondrial fusion. Compared with MSCs isolated from young donors, those derived from aged donors showed a lower level of FGF21 and a higher level of senescent activity. Furthermore, overexpression of FGF21 in aged MSCs inhibited senescence. Our study shows that FGF21, via the AMPK signaling pathway, regulates the senescence of MSCs by mediating mitochondrial dynamics. Targeting FGF21 might represent a novel strategy to improve the quality and quantity of MSCs.

## 1. Introduction

Over the past decades, mesenchymal stem cell- (MSC-) based therapy has emerged as a promising tool for regenerative medicine due to its self-renewal, multiple-lineage differentiation, and immunomodulatory capacity. Although MSCs can be easily obtained from several tissues in the human body, they need to be expanded *in vitro* for several weeks to obtain an adequate amount for clinical trials [1, 2]. Nonetheless, MSCs can undergo only limited cell divisions and prolonged expansion inevitably leads to replicative senescence [3, 4]. This replicative senescence remarkably diminishes their functional and regenerative capacity, thus reducing their therapeutic efficacy [5–7]. The potential mechanisms that underlie the cellular senescence of MSCs remain largely unknown.

Fibroblast growth factor 21 (FGF21), a member of the FGF family, is an endocrine factor that primarily mediates glucose and lipid metabolism. Recently, accumulating evidence has demonstrated that FGF21 plays a critical role in the antiaging process. FGF21 treatment can ameliorate a variety of age-related metabolic diseases, and transgenic mice that overexpress FGF21 have an extended lifespan [8, 9]. Furthermore, FGF21 delays the replicative senescence of endothelial cells by regulating the level of SIRT1 [10]. FGF21 represses angiotensin-induced cellular senescence of human brain vascular smooth muscle cells by regulating mitochondrial biogenesis [11]. Whether and how FGF21 regulates the cellular senescence of MSCs have not been determined.

Reactive oxidative species (ROS) is one of the well-established senescence-triggering mechanisms. A pesticide mixture induces cellular senescence of MSCs by upregulating ROS generation, thus impairing their differentiation capacity [12]. ROS-induced suppression of c-Maf leads to the replicative senescence of MSCs, increasing adipogenic differentiation and reducing osteogenic differentiation [13]. To the best of our knowledge, mitochondria are the major source of ROS. Mitochondria constantly undergo fission and fusion and form a dynamic network to maintain cell function [14]. Mitochondrial fission is mainly regulated by dynamin-related protein 1 (Drp1) and fission-1 (Fis1), and mitochondrial fusion is regulated by Mitofusin1 (Mfn1) and Mitofusin2 (Mfn2) and optic atrophy protein 1 (OPA1). Phosphorylation of Drp1 at Ser616 (p-Drp1 Ser616) increases Drp1 translocation to mitochondria and therefore induces mitochondrial fission [15]. It is well documented that the mitochondrial structure is closely associated with ROS generation and disruption of mitochondrial dynamics results in ROS production with consequent cell injury and cellular senescence [16–20]. Nonetheless, whether FGF21 mediates MSC senescence by regulating mitochondrial dynamics remains to be elucidated.

Given these findings, we hypothesized that FGF21 mediates MSC senescence via regulation of mitochondrial dynamics and ROS production. We show that depletion of FGF21-induced mitochondrial fusion and ROS overproduction lead to senescence of MSCs. Furthermore, FGF21-regulated mitochondrial dynamics are involved in the AMPK signaling pathway.

## 2. Materials and Methods

### 2.1. Cell Culture

Bone marrow (BM) MSCs were cultured with DMEM/high glucose (Gibco) plus 10% FBS (Life Technologies, 16000), 0.1 mM 2-mercaptoethanol (Life Technologies, 21985023), NEAA (Life Technologies, 11140050), penicillin-streptomycin (Life Technologies, 15140122), 5 ng/mL EGF (PeProTech, AF-100-15), and 5 ng/mL FGF (PeProTech, 100-18B) at 37°C in a 5% CO_2_ incubator. MSCs were passaged at a ratio of 1 : 4 when they reached 80%-90% confluence. Human young MSCs and aged MSCs were harvested from volunteer donors at Shanghai East Hospital as previously described [21]. The procedure was approved by the Ethics Committee of Shanghai East Hospital, and written informed consent was obtained from all the volunteers. The passage 4-5 of young MSCs and aged MSCs was used in the current study. Informed consent was obtained from all volunteers. According to age, a total of twenty volunteers were divided into young (young, 18–25 years old) or aged group (aged, 65–80 years old).

### 2.2. Lentiviral Construct Packaging and Infection

Two lentivirus-based recombinant plasmids were constructed. One was inserted by FGF21; the other was inserted by the same backbone as control. The lentiviruses were packaged by transfecting 293T cells using the lentiviral packaging system that comprised the recombinant lentiviral transfer FGF21 plasmid, packaging (GAG/Pol and REV) plasmids, and envelope (VSV-G) plasmid. Following 48 hours of culture, the supernatant of transfecting 293T cells was collected, concentrated, and tittered. Subsequently, the virus was used to infect BM-MSCs. Finally, gene transduction efficiency was evaluated by Western blotting.

### 2.3. Senescence-Associated *β*-Galactosidase (SA-*β*-Gal) Staining

SA-*β*-gal staining was performed according to the manufacturer's protocol (Cell Signaling Technology). Briefly, MSCs were washed with PBS and fixed using the fixative solution for half an hour at room temperature and then incubated at 37°C overnight with the SA-*β*-gal staining solution. The senescent MSCs stained with blue were photographed. The percentage was calculated from five different view fields of each sample in three independent experiments.

### 2.4. Small-Interfering RNA (siRNA) Silencing

FGF21 siRNA (Santa Cruz, sc-39484), Mfn2 siRNA (Santa Cruz, SC-43928), and control siRNA were used to transfect MSCs using a Lipofectamine RNAiMAX Reagent Kit (Invitrogen, 13778-075) at a standardized MOI (multiplicity of infection) of 5 according to the protocol. Seventy-two hours after FGF21 siRNA and Mfn2 siRNA transfection, MSCs were harvested and the silencing efficiency evaluated by Western blotting.

### 2.5. ELISA

The conditioned medium of MSCs at passage 4 and passage 12 was collected, respectively. In brief, a total of 5 × 10^6^ MSCs were plated on a 10 cm culture plate. Twenty-four hours later, the culture medium was changed with 10 mL of serum- and antibiotic-free DMEM. After a further 24 hours, the supernatant was collected gently, filtered through a 0.22 *μ*m filter. The concentration of FGF21 in the conditioned medium was determined by FGF-21 ELISA Kit (DF2100, R&D Systems).

### 2.6. Mdivi-1 Treatment

MSCs were incubated with mitochondrial fission protein Drp1 inhibitor Mdivi-1 (SC-215291, 10 *μ*M) for 24 hours when MSCs reached 60~70% confluence and were then cultured for a further 48 hours and harvested for the following experiments.

### 2.7. Measurement of Mitochondrial ROS

Mitochondrial ROS in MSCs were determined by Mito-Sox staining. Briefly, MSCs were cultured in 24-well plates with glass coverslips with different treatments. Next, MSCs were incubated with 5 *μ*M Mito-Sox (Invitrogen, M36008) for 15 min at 37°C in the dark. Finally, the sample was randomly photographed and the fluorescence intensity analyzed from five different view fields of each group using ImageJ software in three independent experiments.

### 2.8. Western Blotting

The proteins of MSCs with different treatments were extracted and the concentrations measured. A total of 20 *μ*g protein from each sample was loaded, separated by SDS/PAGE, and then transferred to a PVDF membrane. Next, after blocking with 5% fat-free milk in TBST, the membrane was incubated at 4°C overnight with the following antibodies: anti-FGF21 (Abcam, ab171941), anti-p53 (Abcam, ab26), anti-p21 (Abcam, ab109199), anti-Ki-67 (Abcam, ab15580), anti-p-Drp1 ser616 (Invitrogen, PA5-64821), anti-Drp1 (Invitrogen, PA5-20176), anti-Mfn2 (Abcam, ab124773), and GAPDH (Santa Cruz, SC-137179). The membrane was washed with TBST and incubated with horseradish peroxidase-conjugated secondary antibodies (1 : 5000; Santa Cruz) at room temperature for one hour and then proceeded to development.

### 2.9. MitoTracker Staining

The morphology of MSCs was examined by MitoTracker Green FM (Invitrogen, Carlsbad, CA, USA). MSCs given different treatments were incubated for 30 min with DMEM supplemented with 20 nM MitoTracker Green FM. After washing with PBS three times, cells were mounted with DAPI and photographed using a confocal microscope.

### 2.10. Statistical Analysis

All data are presented as the mean ± SEM. Statistical analyses were performed using Prism 5.04 software (GraphPad Software for Windows, San Diego, CA, USA). Comparison between two groups was analyzed by unpaired Student's *t*-test and comparison between multiple groups by one-way ANOVA followed by the Bonferroni test. A *p* value < 0.05 was considered statistically significant.

## 3. Results

### 3.1. Expansion of MSCs Induces Cellular Senescence *In Vitro*

**Figure 1:**
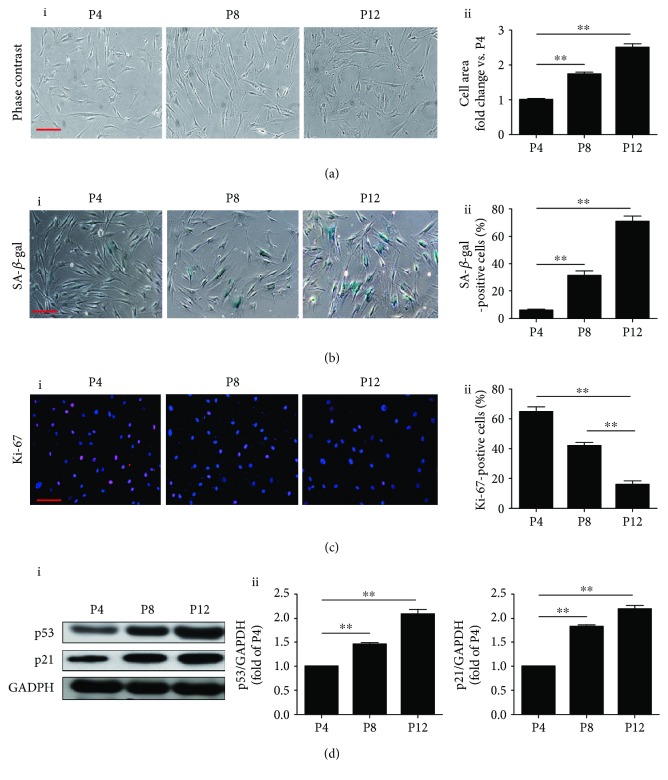
Characterization of BM-MSCs replicative senescence. (a) Representative images of BM-MSC morphology under light microscopy at passage 4 (P4), passage 8 (P8) and passage 12 (P12) (i). The cell size of MSCs was calculated at P4, P8, and P12 (ii). (b) Representative images of SA-*β*-gal staining in BM-MSCs at P4, P8, and P12 (i). The SA-*β*-gal-positive cells at P4, P8, and P12 were calculated and presented as percentage of the total cells (ii). (c) Representative images of Ki-67 staining in BM-MSCs at P4, P8, and P12 (i). The Ki-67-positive cells at P4, P8, and P12 were calculated and presented as percentage of the total cells (ii). (d) Western blotting and quantitative analysis of the level of p53 and p21 proteins in BM-MSCs at P4, P8, and P12 (i, ii). Data are expressed as mean ± SEM with *n* = 3 per group. ^∗∗^ *p* < 0.01. Scale bar = 100 *μ*m.

We first examined the morphology of MSCs at different passages under light microscopy. MSCs at passage 4 (P4) showed a healthy spindle shape but an enlarged and flattened shape in passage 8 (P8) and in passage 12 (P12) (Figure 1(a), i). Compared with MSCs in P4, the cell size was significantly increased in P8 and P12 (Figure 1(a), ii). Next, the senescence of MSCs at different passages was examined using SA-*β*-gal staining. The percentage of SA-*β*-gal-positive cells was greatly enhanced in P8 and P12 compared with P4 cells (Figure 1(b), i and ii). To further validate our observation, Ki-67 staining was performed to examine the proliferative capacity of MSCs at different passages. There was a significant decrease in the percentage of Ki-67-positive MSCs from P4 to P12 (Figure 1(c), i and ii). We also examined the level of cellular senescence-related proteins p53 and p21. Levels of both were markedly elevated in P8 and P12 BM-MSCs compared with those in P4 (Figure 1(d)). We conclude that BM-MSCs can develop a senescent phenotype during consecutive passaging expansion.

### 3.2. Depletion of FGF21 Induces Cellular Senescence of MSCs

**Figure 2:**
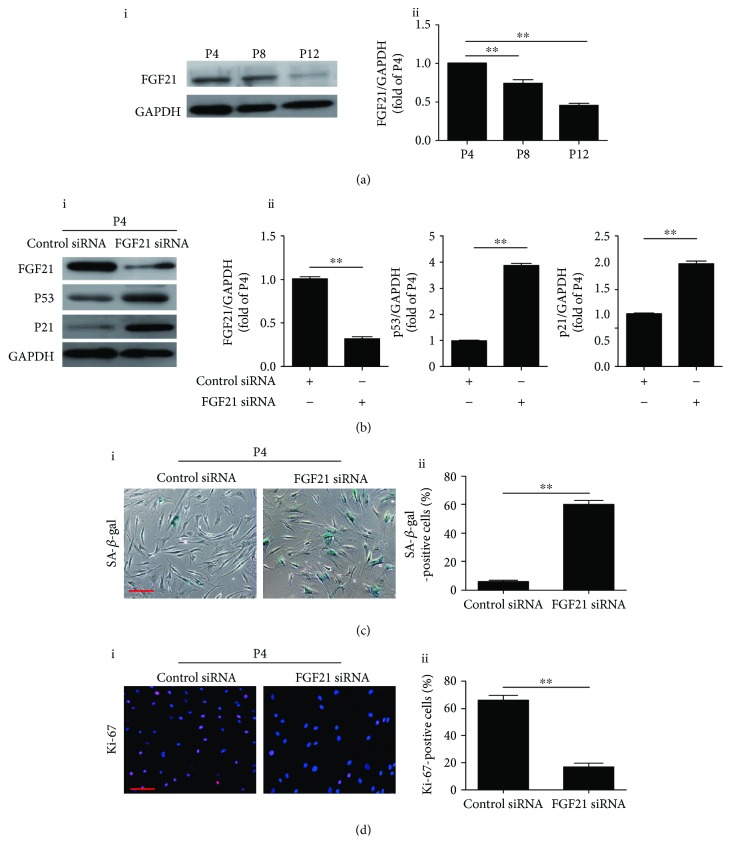
FGF21 mediates the replicative senescence of BM-MSCs. (a) Western blotting and quantitative analysis of the level of FGF21 protein in BM-MSCs at P4, P8, and P12 (i, ii). (b) Western blotting and quantitative analysis of the level of FGF21, p53, and p21 proteins in control siRNA or FGF21 siRNA-treated BM-MSCs at P4 (i, ii). (c) Representative images of SA-*β*-gal staining in control siRNA or FGF21 siRNA-treated BM-MSCs at P4 (i). Percentage of SA-*β*-gal-positive senescent cells in control siRNA or FGF21 siRNA-treated-BM-MSCs at P4 (ii). (d) Representative images of Ki-67 staining in control siRNA or FGF21 siRNA-treated BM-MSCs at P4 (i). Percentage of Ki-67-positive senescent cells in control siRNA or FGF21 siRNA-treated BM-MSCs at P4 (ii). Data are expressed as mean ± SEM with *n* = 3 per group. ^∗∗^ *p* < 0.01. Scale bar = 100 *μ*m.

To determine the effects of FGF21 on cellular senescence of MSCs, we examined the expression level of FGF21 at different passages. Western blotting showed that FGF21 was gradually reduced in a passage-dependent manner, suggesting that FGF21 may be involved in regulating the senescence of MSCs (Figure 2(a), i and ii). Furthermore, compared with P4 BM-MSCs, the concentration of FGF21 in the conditioned medium at P12 BM-MSCs was greatly reduced (128.3 ± 7.8 pg/mL vs. 50.7 ± 5.9 pg/mL), indicating that MSCs secrete FGF21 and the concentration declines during consecutive passaging expansion. To verify whether decreased FGF21 is responsible for inducing senescence of MSCs, we treated P4 BM-MSCs with FGF21 siRNA. We found that FGF21 siRNA treatment greatly reduced the protein level of FGF21 but upregulated the p53 and p21 protein level in P4 BM-MSCs (Figure 2(b), i and ii). FGF21 siRNA treatment also markedly enhanced SA-*β*-gal-positivity in P4 BM-MSCs (Figure 2(c), i and ii). Furthermore, Ki-67 staining revealed that the number of Ki-67-positive cells was significantly reduced in FGF21 siRNA-treated P4 BM-MSCs compared with control BM-MSCs (Figure 2(d), i and ii). Taken together, these results reveal that knockdown of FGF21 induces cellular senescence in early-passage BM-MSCs.

### 3.3. FGF21 Knockdown Induces Senescence via ROS Generation

**Figure 3:**
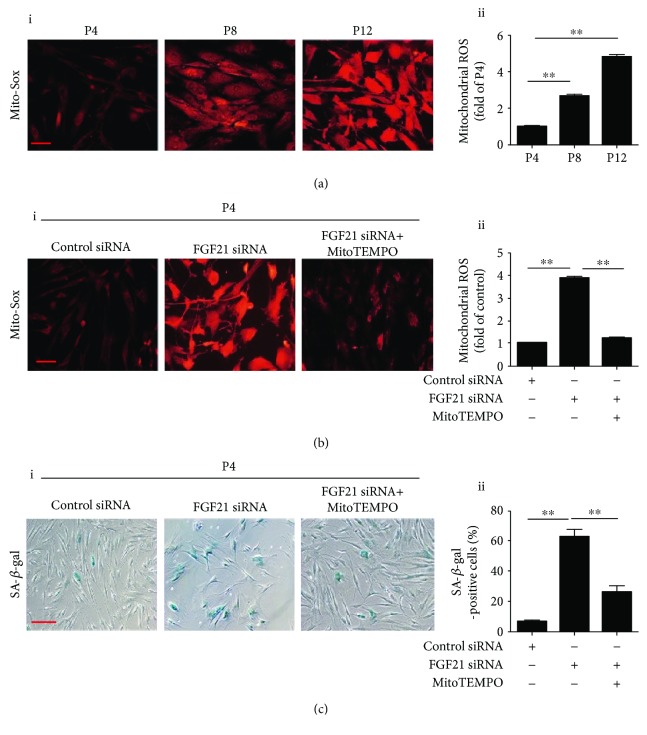
Silencing FGF21 induces MSC senescence via ROS generation. (a) Representative images of Mito-Sox staining in BM-MSCs at P4, P8, and P12 (i). Quantitative analysis of ROS generation in BM-MSCs at P4, P8, and P12 (ii). (b) Representative images of Mito-Sox staining in BM-MSCs at P4 from the control siRNA or FGF21 siRNA or FGF21 siRNA+MitoTEMPO-treated group (i). Quantitative analysis of ROS generation in control siRNA or FGF21 siRNA or FGF21 siRNA+MitoTEMPO-treated BM-MSCs at P4 (ii). (c) Representative images of SA-*β*-gal staining in BM-MSCs at P4 from the control siRNA or FGF21 siRNA or FGF21 siRNA+MitoTEMPO-treated group (i). Percentage of SA-*β*-gal-positive senescent cells in control siRNA or FGF21 siRNA or FGF21 siRNA+MitoTEMPO-treated BM-MSCs at P4 (ii). Data are expressed as mean ± SEM with *n* = 3 per group. ^∗∗^ *p* < 0.01. Scale bar = 100 *μ*m.

It is well established that ROS from mitochondria plays a very important role in regulating cellular senescence. We therefore used Mito-Sox staining to detect ROS production at different passages of BM-MSCs. As shown in Figure 3(a), the ROS level was significantly enhanced in P8 and P12 BM-MSCs compared with that in P4 (Figure 3(a), i and ii), suggesting that ROS may be involved in the regulation of MSC senescence. FGF21 siRNA treatment markedly enhanced ROS generation in P4 BM-MSCs (Figure 3(b), i and ii). Nonetheless, treatment with MitoTEMPO, a mitochondrial ROS inhibitor, significantly inhibited FGF21 siRNA-induced ROS generation in P4 BM-MSCs (Figure 3(b), i and ii). More importantly, MitoTEMPO treatment also dramatically reduced FGF21 siRNA-induced senescence of P4 BM-MSCs (Figure 3(c), i and ii)). Collectively, our results reveal that FGF21 knockdown induces senescence of MSCs by increasing ROS generation.

### 3.4. FGF21 Knockdown Induces ROS Generation via Regulation of Mitochondrial Dynamics

**Figure 4:**
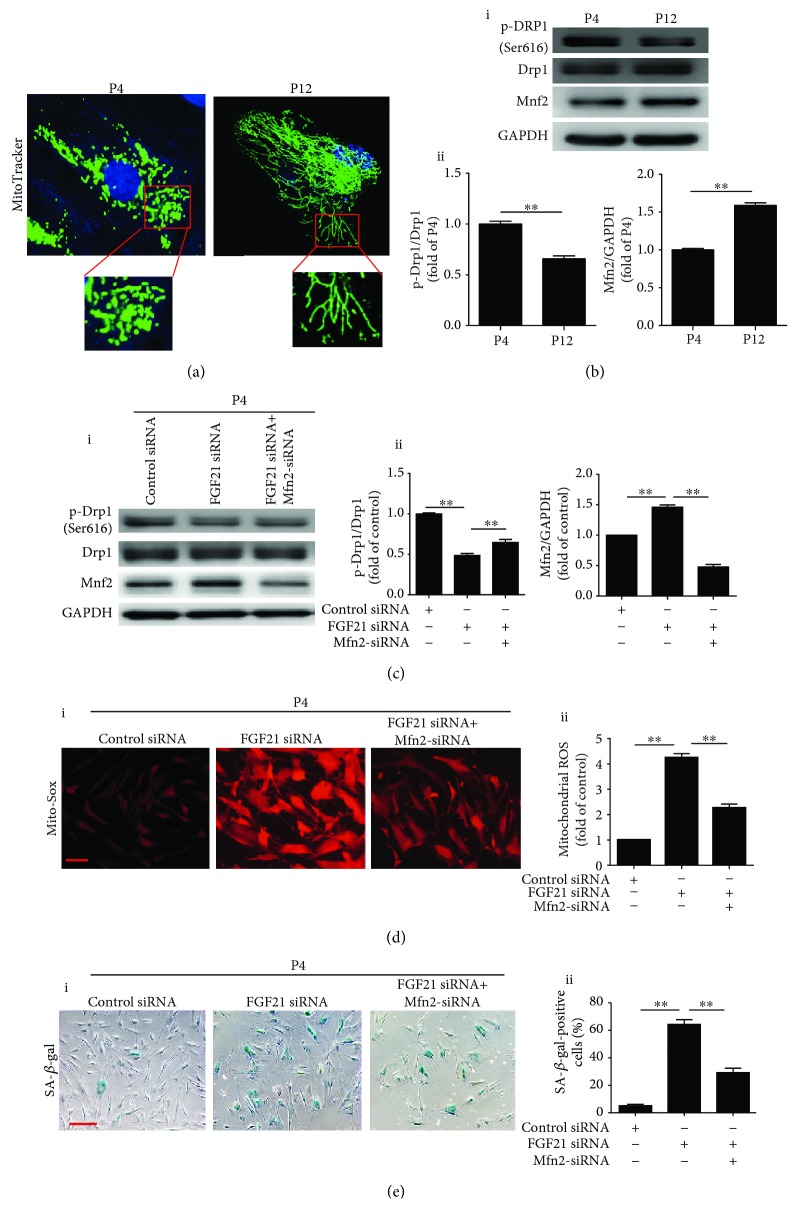
Silencing FGF21 induces ROS generation via regulation of mitochondrial dynamics. (a) Representative images of MitoTracker staining in BM-MSCs at P4 and P12. (b) Western blotting and quantitative analysis of the levels of p-Drp1/Drp1 and Mfn2 proteins in BM-MSCs at P4 and P12 (i, ii). (c) Western blotting and quantitative analysis of the level of p-Drp1/Drp1 and Mfn2 proteins in control siRNA or FGF21 siRNA or FGF21 siRNA+Mfn2 siRNA-treated BM-MSCs at P4 (i, ii). (d) Representative images of Mito-Sox staining in BM-MSCs at P4 from the control siRNA or FGF21 siRNA or FGF21 siRNA+Mfn2 siRNA-treated group (i). Quantitative analysis of the ROS generation in control siRNA or FGF21 siRNA or FGF21 siRNA+Mfn2 siRNA-treated BM-MSCs at P4 (ii). (e) Representative images of SA-*β*-gal staining in BM-MSCs at P4 from the control siRNA or FGF21 siRNA or FGF21 siRNA+Mfn2 siRNA-treated group (i). Percentage of SA-*β*-gal-positive senescent cells in control siRNA or FGF21 siRNA or FGF21 siRNA+Mfn2 siRNA-treated BM-MSCs at P4 (ii). Data are expressed as mean ± SEM with *n* = 3 per group. ^∗∗^ *p* < 0.01. Scale bar = 100 *μ*m.

Accumulating evidence has demonstrated that mitochondrial fusion and fission are closely related to ROS generation. We first examined the mitochondrial morphology of BM-MSCs during P4 and P12. MitoTracker green staining demonstrated small tubular mitochondria in P4 BM-MSCs but large tubular mitochondria in P12 BM-MSCs, indicating that mitochondrial fusion may contribute to cellular senescence (Figure 4(a)). Western blotting showed that compared with P4 BM-MSCs, Mfn2 was greatly enhanced whereas p-Drp1 was markedly reduced in P12 BM-MSCs (Figure 4(b), i and ii). Furthermore, no significant difference of the protein level of Fis1, Mfn1, and OPA1 was observed between P4 BM-MSCs and P12 BM-MSCs (Figure S1). Next, we treated P4 BM-MSCs with Mdivi-1 for 24 hours and cultured them for a further 48 hours. Western blotting showed that Mdivi-1 treatment enhanced the protein level of Mfn2 and reduced the level of p-Drp1, suggesting the occurrence of mitochondrial fusion (Figure S2A-i, ii). Furthermore, Mdivi-1 treatment significantly increased mitochondrial ROS generation (Figure S2B-i, ii) and enhanced SA-*β*-gal positivity of P4 BM-MSCs (Figure S2C-i, ii), indicating that mitochondrial fusion induces senescence of BM-MSCs. To further illustrate the relationship between FGF21 and mitochondrial dynamics, P4 BM-MSCs were transfected with FGF21 siRNA. Silencing FGF21 with FGF21 siRNA elevated the level of Mfn2 and decreased the level of p-Drp1 (Figure 4(c)). Meanwhile, ROS generation and senescent activity were enhanced in FGF21 siRNA-treated P4 BM-MSCs (Figures 4(d) and 4(e)). On the contrary, Mfn2 siRNA treatment attenuated the increased expression of Mfn2 and decreased the expression of p-Drp1 in FGF21 siRNA-treated P4 BM-MSCs (Figure 4(c)). Silencing Mfn2 also abrogated FGF21 siRNA-induced ROS generation and SA-*β*-gal activity (Figures 4(d) and 4(e)). Collectively, we conclude that FGF21 regulates the senescence of BM-MSCs via the mediation of mitochondrial dynamics.

### 3.5. FGF21 Regulates Mitochondrial Dynamics by Mediating AMPK Activation

**Figure 5:**
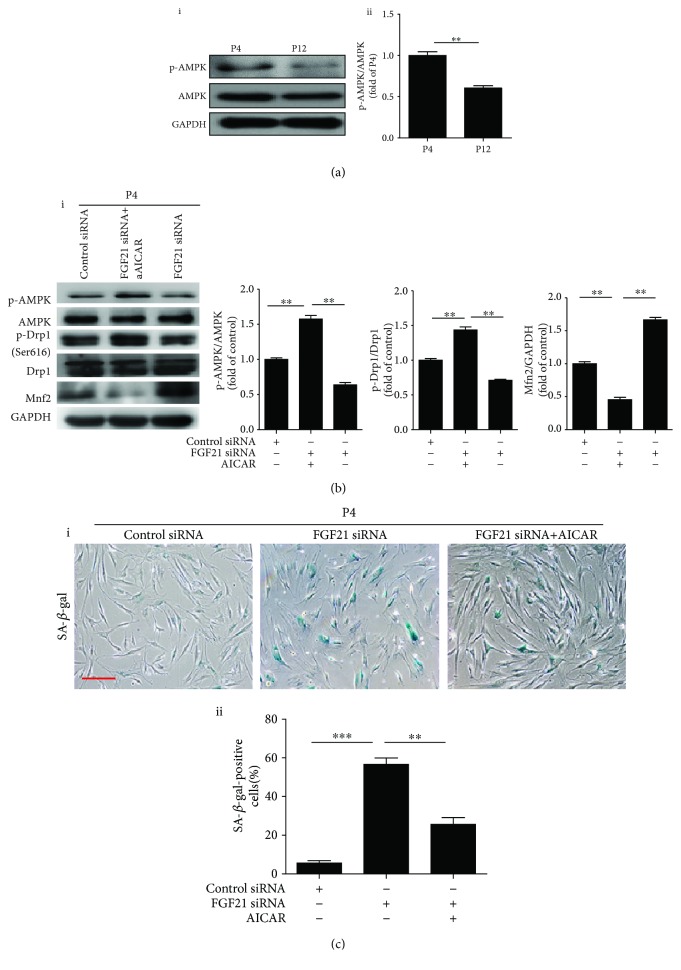
FGF21 regulates mitochondrial dynamics via mediation of AMPK activation. (a) Western blotting and quantitative analysis of the level of p-AMPK in BM-MSCs at P4 and P12 (i, ii). (b) Western blotting and quantitative analysis of the level of p-AMPK, p-Drp1, and Mfn2 in BM-MSCs at P4 from the control siRNA or FGF21 siRNA or FGF21 siRNA+AICAR-treated group (i, ii). (c) Representative images of SA-*β*-gal staining in BM-MSCs at P4 from the control siRNA or FGF21 siRNA or FGF21 siRNA+AICAR-treated group (i). Percentage of SA-*β*-gal-positive senescent cells in control siRNA or FGF21 siRNA or FGF21 siRNA+AICAR-treated BM-MSCs at P4 (ii). Data are expressed as mean ± SEM with *n* = 3 per group. ^∗^ *p* < 0.05, ^∗∗^ *p* < 0.01. Scale bar = 100 *μ*m.

Previous studies have demonstrated that AMPK regulates mitochondrial structure/function and dynamics [22, 23]. We attempted to determine whether FGF21 regulates mitochondrial dynamics via mediation of AMPK. First, we examined AMPK activation in different passages of MSCs. Western blotting showed that AMPK phosphorylation (p-AMPK) was gradually reduced in MSCs during consecutive passaging expansion (Figure 5(a), i and ii). To determine whether AMPK activation is responsible for mitochondrial dynamics, we treated P4 BM-MSCs with compound C, an AMPK inhibitor. Compound C treatment significantly upregulated Mnf2 protein but downregulated p-Drp1 (Figure S3A-i, ii). Furthermore, compound C treatment significantly enhanced the senescence of MSCs at P4 (Figure S3B-i, ii). These results suggest that the AMPK pathway may participate in regulation of MSC senescence by mediating mitochondrial dynamics. Subsequently, we examined whether FGF21 mediates mitochondrial dynamics by regulating AMPK. Western blotting analysis showed that silencing FGF21 with FGF21 siRNA reduced p-AMPK and p-Drp1 and upregulated the Mfn2 level (Figure 5(b)) in P4 BM-MSCs. Nonetheless, the AMPK activator, AICAR, enhanced the reduced p-AMPK and p-Drp1 and decreased the upregulated Mfn2 in FGF21 siRNA-treated MSCs (Figure 5(b)). Furthermore, AICAR abrogated FGF21 silence-induced MSC senescence (Figure 5(c)). To further verify whether FGF21 regulates mitochondrial dynamics by activating AMPK, we overexpressed FGF21 in P12 BM-MSCs. Western blotting showed that overexpressed FGF21 in P12 BM-MSCs greatly enhanced the protein level of p-AMPK and p-Drp1 but reduced Mfn2 (Figure S4). Furthermore, these effects were partly reversed by the AMPK inhibitor, compound C (Comp C) (Figure S4). These results indicate that FGF21 regulates mitochondrial dynamics via the AMPK signaling pathway and in turn mediates cell senescence.

### 3.6. Overexpression of FGF21 in Aged MSCs Attenuates Cell Senescence

**Figure 6:**
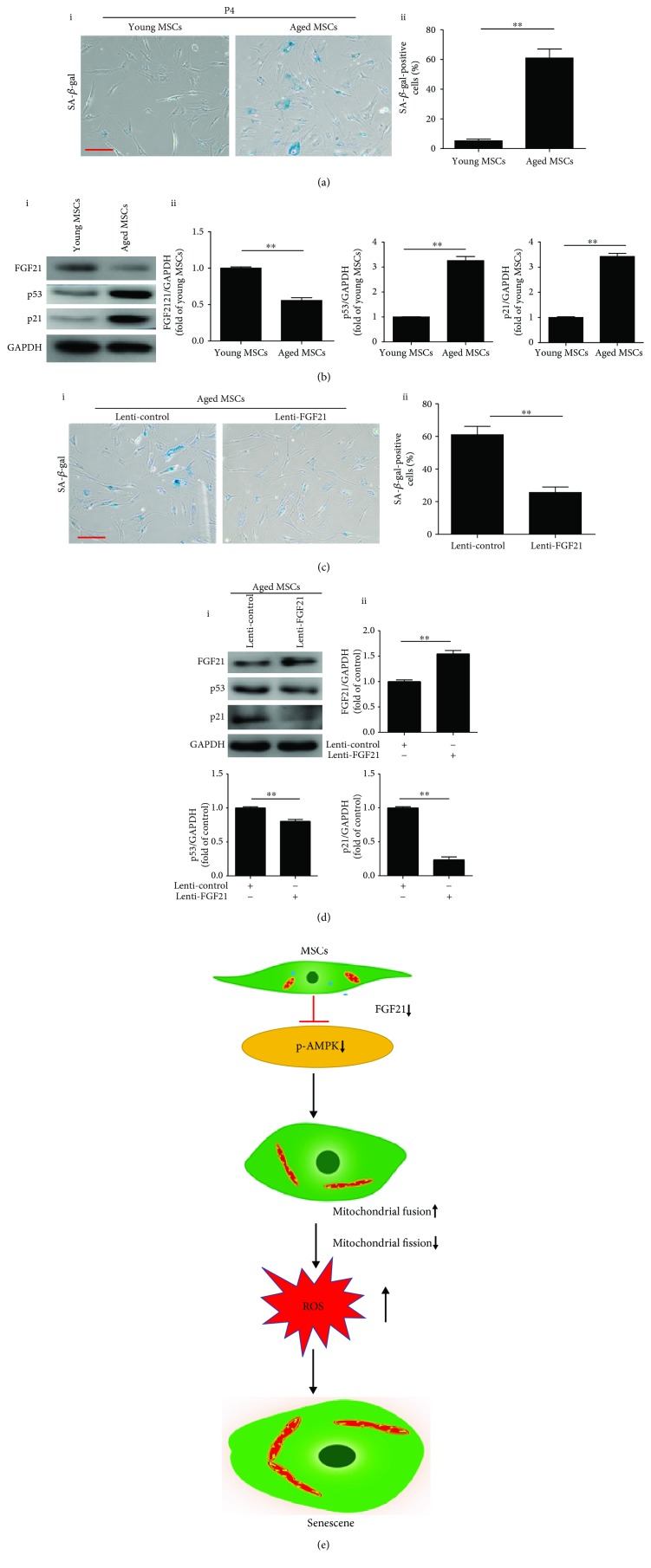
Overexpression of FGF21 in aged MSCs reduces cell senescence. (a) Representative images of SA-*β*-gal staining in BM-MSCs isolated from young and aged donors (i). Percentage of SA-*β*-gal-positive senescent cells in young MSCs and aged MSCs (ii). (b) Western blotting and quantitative analysis of the level of FGF21, p53, and p21 in young MSCs and aged MSCs (i, ii). (c) Representative images of SA-*β*-gal staining in aged BM-MSCs transfected with control lentivirus or FGF21 lentivirus (i). Percentage of SA-*β*-gal-positive senescent cells in aged BM-MSCs transfected with control lentivirus or FGF21 lentivirus (ii). (d) Western blotting and quantitative analysis of the level of FGF21, p53, and p21 in aged BM-MSCs transfected with control lentivirus or FGF21 lentivirus (i, ii). (e) The proposed mechanisms involved in FGF21 mediates MSC senescence via regulation of mitochondrial dynamics. Data are expressed as mean ± SEM with *n* = 3 per group. ^∗∗^ *p* < 0.01. Scale bar = 100 *μ*m.

To determine whether FGF21 mediates the physiological senescence of MSCs, we isolated MSCs from young and aged donors and examined the cellular senescence. Compared with young MSCs, aged MSCs demonstrated a decreased level of FGF21 and increased protein level of p53 and p21 as well as activity of SA-*β*-gal (Figures 6(a) and 6(b)). On the contrary, overexpression of FGF21 in aged MSCs dramatically reduced the activity of SA-*β*-gal (Figure 6(c), i and ii). Furthermore, overexpression of FGF21 enhanced the level of FGF21 and reduced the levels of p53 and p21 proteins in aged MSCs (Figure 6(d), i and ii). These observations support the notion that FGF21 also regulates the physiological senescence of MSCs.

## 4. Discussion

There are some major findings in the current study (Figure 6(e)). First, the level of FGF21 was significantly reduced in MSCs with increased passage and in MSCs isolated from aged donors. Second, disruption of mitochondrial dynamics and induced ROS production resulted in senescence of MSCs. Third, FGF21 mediated MSC senescence via regulation of mitochondrial dynamics. Last but not least, the AMPK signaling pathway was involved in FGF21 regulation of mitochondrial dynamics.

Although MSC-based therapy has shown promising therapeutic potential in a variety of disorders in preclinical studies and clinical trials [24–26], MSCs can easily become senescent even under standard culture conditions as the passage increases *in vitro*, thus reducing their beneficial effects [27, 28]. Senescent MSCs can release a variety of factors including inflammatory cytokines, termed senescence-associated secretory phenotype (SASP). These factors further exacerbate cellular senescence and reduce the function of MSCs; thus, a vicious circle begins [29]. The senescent MSCs exhibit growth arrest, enlarged cell size, increased lysosomal content characterized by SA-*β*-gal, and lower proliferative capacity [30]. The activity of p53, by inducing a downstream gene p21, is positively correlated with cellular senescence [31]. In this study, we showed that BM-MSCs developed into the senescent phenotype during consecutive passaging expansion as evidenced by an increased cell size, a lower level of Ki-67, enhanced SA-*β*-gal positivity, and elevation of the p53 and p21 level.

Since BM-MSCs show replicative senescence in long-term culture, elucidating the potential mechanisms that underlie MSC senescence may help provide novel therapeutic strategies to delay senescence. An increasing number of studies have shown that FGF21 is a very important regulator in controlling longevity. Compared with wild-type mice, transgenic mice that overexpress FGF21 live longer with no change in food intake [32]. Furthermore, FGF21 reduces immune senescence by inhibiting age-related thymic involution [33]. These observations suggest that FGF21 has a strong antiaging capacity. Indeed, in the current study, we found that MSCs displayed a decreased level of FGF21 that was accompanied by an increased level of p53 and p21 during the passaging process, indicating that FGF21 may participate in the regulation of the replicative senescence of MSCs. Furthermore, knockdown of FGF21 using siRNA in the early passage of MSCs reinforced the senescence. On the contrary, overexpression of FGF21 in aged MSCs ameliorated the senescence. These findings are further evidence that FGF21 regulates both the physiological and replicative senescence of MSCs.

Despite thorough investigation, the molecular network underlying MSC senescence is not fully understood. Several mechanisms including telomere shortening, autophagy, and especially increased ROS have been well documented in MSC senescence [6, 34, 35]. Monocyte chemoattractant protein-1 (MCP-1) reinforces the senescence of MSCs derived from umbilical cord blood via activation of the ROS-p38-MAPK-p53/p21 signaling pathway [36]. In accordance with previous studies [1, 37], our results also showed that the senescent MSCs (P8, P12) exhibited an increased level of ROS. Knockdown of FGF21 in the early passage of MSCs enhanced ROS generation whereas ROS scavengers significantly prevented the senescence, indicating that knockdown of FGF21 induces senescence of MSCs by inducing ROS generation. ROS production is mainly mediated by the imbalance between mitochondrial fusion and fission [16, 38]. Depletion of protein disulfide isomerase A1 (PDIA1) promotes mitochondrial fission and elevates ROS generation and finally induces endothelial cell senescence [39]. In contrast, downregulation of Drp1 inducing mitochondrial fusion contributes to endothelial cell senescence by enhancing ROS production [40]. Moreover, the senescent MSCs isolated from human adipose tissue exhibit increased mitochondrial fusion and enhanced ROS production [37]. These contrary results may be due to the different types of cells and different stresses that caused cellular senescence. In this study, we found enhanced mitochondrial fusion as evidenced by elongated mitochondria and enhanced Mfn2 and decreased p-Drp1 protein P12 BM-MSCs compared with P4 BM-MSCs. Inhibition of mitochondrial fission using Mdivi-1 dramatically enhanced mitochondrial ROS production and increased MSC senescence. More importantly, we also showed that knockdown of FGF21 in the early passage of MSCs greatly elevated mitochondrial fusion as evidenced by the enhanced Mfn2 and decreased p-Drp1. Nevertheless, knockdown of Mfn2 reversed FGF21 knockdown-induced mitochondrial fusion and senescence of MSCs. Based on the above results, we conclude that FGF21 mediates MSC senescence via regulation of mitochondrial dynamics.

Adenosine monophosphate-activated protein kinase (AMPK) is a conserved, redox-activated master regulator of cell metabolism. Recently, an increasing number of studies have shown that AMPK signaling is essential to maintain mitochondrial dynamics and function [41, 42]. AMPK activation enhances mitochondrial fission via phosphorylation of mitochondrial fission factor (MFF) and ULK1 [43, 44]. FGF21 is closely related to AMPK activation [45]. FGF21 extends the mammalian lifespan via activation of AMPK [9]. Moreover, the protective effects of FGF21 on cardiomyopathy have been attributed to activation of AMPK [46, 47]. Given the relationship between FGF21 and AMPK, we next addressed whether FGF21 regulation of mitochondrial dynamics is via mediation of AMPK activation. We showed that knockdown of FGF21 significantly reduced AMPK activation and enhanced mitochondrial fusion. In contrast, treatment with AICAR abrogated these effects and reduced knockdown of FGF21-induced cellular senescence.

Several limitations of the current study need to be highlighted. First, we used siRNA to knock down FGF21. It would better illustrate the role of FGF21 in the regulation of cell senescence if we had used Crispr/Cas9 to knock out FGF21. Second, the potential role of telomere shortening or autophagy in FGF21-mediated MSC senescence was not studied. Third, our study used only BM-MSCs. Whether these findings can be applied to MSCs derived from other tissues such as adipose and umbilical cord remains to be investigated. Last but not least, the mechanisms underlying FGF21 regulating the AMPK signal pathway still remain unclear.

## 5. Conclusion

In summary, our study demonstrates that FGF21 regulates the senescence of MSCs via mediation of mitochondrial dynamics and ROS production in an AMPK-dependent manner. FGF21 may be a promising target for rejuvenating the cellular senescence of MSCs.

## Data Availability

All data used in the current study are available from the corresponding author upon request.

## Supplementary Materials

Supplementary Materials Figure S1: the levels of mitochondrial dynamics-related proteins in BM-MSCs at P4 and P12. Figure S2: inhibition of mitochondrial fission induces cell senescence. Figure S3: inhibition of AMPK induces cell senescence via regulating mitochondrial dynamics. Figure S4: overexpression of FGF21 regulates mitochondrial dynamics via activating AMPK in P12 BM-MSCs.
