# Supplementary material for: FGF21 Mediates Mesenchymal Stem Cell Senescence via Regulation of Mitochondrial Dynamics
Source: Oxid Med Cell Longev. 2019 Apr 17;2019:4915149. doi: 10.1155/2019/4915149 (PMC6501200; doi:10.1155/2019/4915149)
Supplement: Supplementary Materials — Figure S1: the levels of mitochondrial dynamics-related proteins in BM-MSCs at P4 and P12. Figure S2: inhibition of mitochondrial fission induces cell senescence. Figure S3: inhibition of AMPK induces cell senescence via regulating mitochondrial dynamics. Figure S4: overexpression of FGF21 regulates mitochondrial dynamics via activating AMPK in P12 BM-MSCs. [file 4915149.f1.docx]

**SUPPORTING INFORMATION**

**
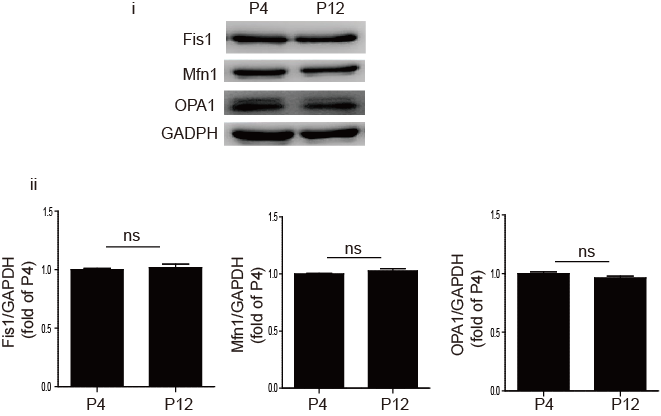
**

**Figure S1. The levels of mitochondrial dynamics-related proteins in BM-MSCs at P4 and P12.** Western blotting and quantitative analysis of the levels of Fis1, Mfn1 and OPA1 proteins in BM-MSCs at P4 and P12 (i, ii). Data are expressed as mean±SEM. ns, not significant.

**
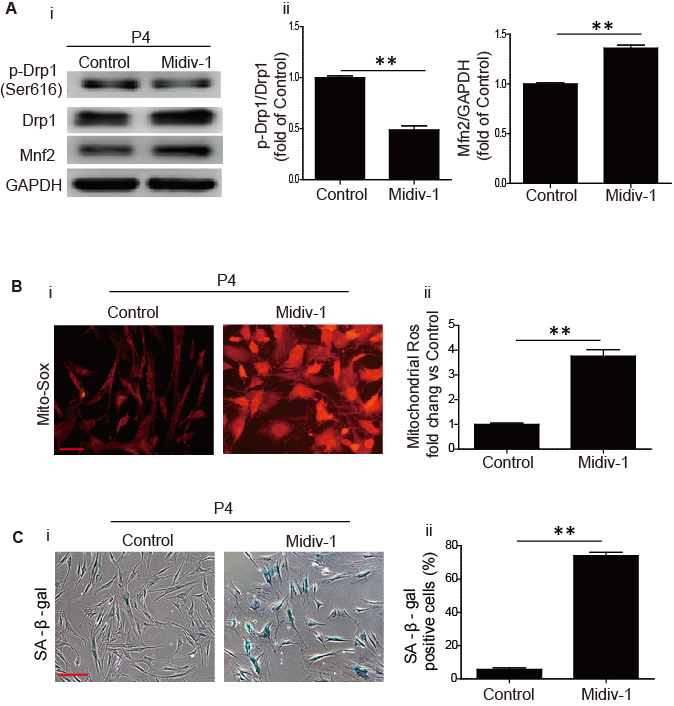
**

**Figure S2. Inhibition of mitochondrial fission induces cell senescence**

(A) Western blotting and quantitative analysis of the level of p-Drp1/Drp1 and Mfn2 proteins in BM-MSCs or Midiv-1-treated BM-MSCs at P4 (i, ii). (B) Representative images of Mito-Sox staining in BM-MSCs or Midiv-1-treated BM-MSCs at P4 (i). Quantitative analysis of the ROS generation in BM-MSCs or Midiv-1-treated BM-MSCs at P4 (ii). (C) Representative images of SA-β-gal staining in BM-MSCs or Midiv-1-treated BM-MSCs at P4 (i). Percentage of SA-β-gal positive senescent cells in BM-MSCs or Midiv-1-treated BM-MSCs at P4 (ii). Data are expressed as mean±SEM. **p < 0.01. Scale bar=100µm

**
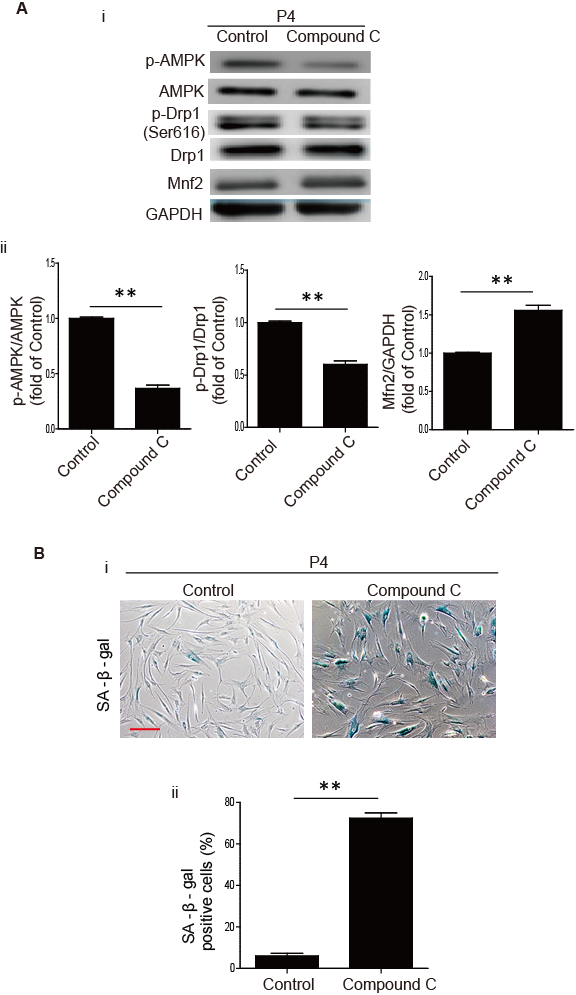
**

**Figure S3. Inhibition of AMPK induces cell senescence via regulating mitochondrial dynamics**

(A) Western blotting and quantitative analysis of the level of p-AMPK/AMPK, p-Drp1/Drp1 and Mfn2 proteins in BM-MSCs or Compound C-treated-BM-MSCs at P4 (i, ii). (B) Representative images of SA-β-gal staining in BM-MSCs or Compound C-treated-BM-MSCs at P4 (i). Percentage of SA-β-gal positive senescent cells in BM-MSCs or Compound C-treated BM-MSCs at P4 (ii). Data are expressed as mean±SEM. ***p < 0.01*. Scale bar=100µm.


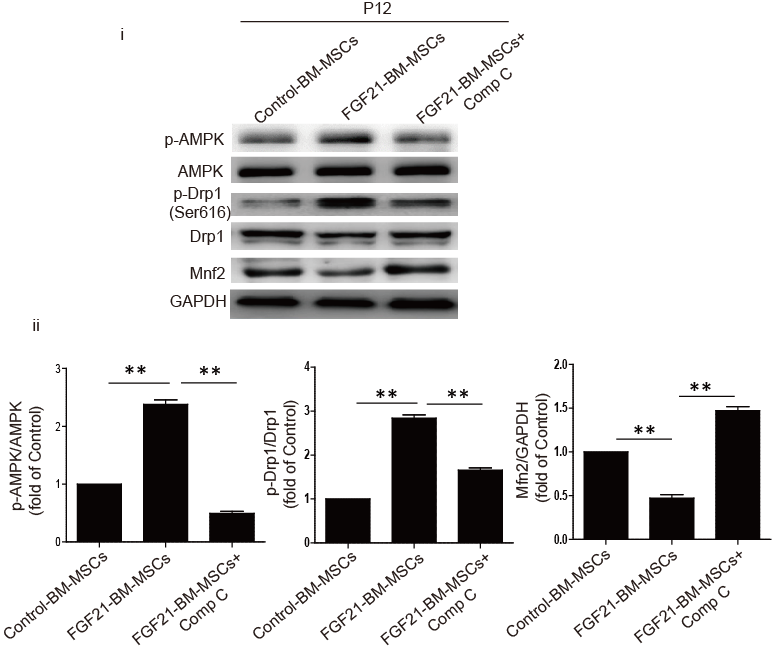


**Figure S4. Overexpression of FGF21 regulates mitochondrial dynamics via activating AMPK in P12 BM-MSCs.** Western blotting and quantitative analysis of the level of p-AMPK/AMPK, p-Drp1/Drp1 and Mfn2 proteins in BM-MSCs or FGF21-BM-MSCs or Compound C-treated-FGF21-BM-MSCs at P12 (i, ii). Data are expressed as mean±SEM. ***p < 0.01*.
